# Microbiological assessment of sheep lymph nodes with lymphadenitis found during *post*-*mortem* examination of slaughtered sheep: implications for veterinary-sanitary meat control

**DOI:** 10.1186/s13028-020-00547-x

**Published:** 2020-09-04

**Authors:** Anna Didkowska, Piotr Żmuda, Ewelina Kwiecień, Magdalena Rzewuska, Daniel Klich, Monika Krajewska-Wędzina, Lucjan Witkowski, Monika Żychska, Aleksandra Kaczmarkowska, Blanka Orłowska, Krzysztof Anusz

**Affiliations:** 1 Department of Food Hygiene and Public Health Protection, Institute of Veterinary Medicine, Warsaw University of Life Sciences “SGGW”, Nowoursynowska 159, 02-776 Warsaw, Poland; 2 University Centre of Veterinary Medicine UJ-UR, Al. Mickiewicza 24/28, 30-059 Kraków, Poland; 3 Department of Preclinical Sciences, Institute of Veterinary Medicine Warsaw, University of Life Sciences–SGGW, Ciszewskiego 8, 02-786 Warsaw, Poland; 4 Institute of Animal Sciences, University of Life Sciences–SGGW, Ciszewskiego 8, 02-786 Warsaw, Poland; 5 National Reference Laboratory for Bovine Tuberculosis, Department of Microbiology, National Veterinary Research Institute, Partyzantow 57, Puławy, Poland; 6 Laboratory of Veterinary Epidemiology and Economic, Institute of Veterinary Medicine, Warsaw University of Life Sciences–SGGW, Nowoursynowska 159c, 02-776 Warsaw, Poland

**Keywords:** Lymph node, Pathogens, *Post*-*mortem* examination, Public health, Purulent and caseous lesions, Sheep

## Abstract

**Background:**

Microbiological examination of lesions found in slaughtered animals during meat inspection is an important part of public health protection as such lesions may be due to zoonotic agents that can be transmitted by meat. Examination of inflamed lymph nodes also plays a particular important role, as lymphadenitis may reflect a more widespread infection. Such lesions in sheep are mainly caused by pyogenic bacteria but also mycobacteria are occasionally found. Meat inspection data from 2017 to 2018 from southern Poland, especially from the Małopolska region, indicate that purulent or caseous lymphadenitis involving the mediastinal and tracheobronchial lymph nodes (MTLNs) is a common finding. The primary aim of the current study was to determine the aetiology of these lesions. Furthermore, it was investigated how presence of lesions was correlated with age and grazing strategy of affected sheep.

**Results:**

*Post*-*mortem* examination revealed purulent or caseous lymphadenitis in the MTLNs of 49 out of 284 animals (17.3%). Subsequent microbiological examination revealed the presence of *Corynebacterium pseudotuberculosis* (34.7%), *Streptococcus dysgalactiae* subsp. *equisimilis* (34.7%), *Staphylococcus aureus* (8.2%), *Enterococcus* spp. (2.0%), *Trueperella pyogenes* (2.0%), and β-haemolytic strains of *Escherichia coli* (2.0%). *Mycobacterium* spp. and *Rhodococcus equi* were not detected. In older sheep, the probability of the presence of purulent or caseous lymphadenitis was higher than in younger, and the risk was increasing by 1.5% with each month of life. Sheep grazing locally had 4.5-times greater risk of having purulent or caseous lymphadenitis than individuals summer grazing in the mountains.

**Conclusion:**

The most common aetiological agents of purulent or caseous lymphadenitis in the MTLNs of sheep in the Małopolska region were *C. pseudotuberculosis* and *S. dysgalactiae* subsp. *equisimilis*. Particular attention during *post*-*mortem* examination should be paid to the carcasses of older sheep and sheep grazing on permanent pastures, as they seem more prone to develop purulent or caseous lymphadenitis.

## Background

Microbiological examination of lesions found in slaughtered animals during meat inspection is an important part of public health protection as such lesions may be due to zoonotic agents, which may be transmitted e.g. by meat contaminated with viable bacteria. In recent decades, a growing number of reports on human diseases caused by bacteria of animal origin have been published [1–3]. For some zoonotic agents such as *Salmonella*, Commission Regulation (EC) No 2073/2005 of 15 November 2005 on microbiological criteria for foodstuff specifies obligatory examinations [4]. However, for those, and other zoonotic pathogens such as *Corynebacterium pseudotuberculosis*, scientific studies are underway to assess the risk for consumers [5–7].

Examination of inflamed lymph nodes plays a particular important role. Presence of lymphadenitis may reflect not only a localized infection but also infection of their drainage bed thus indicating a more widespread infection. Also, some bacterial species, of which some are zoonotic, localize to the lymph nodes and induces rather characteristic lesions such as granulomatous inflammation. The differential aetiology of purulent lymphadenitis in sheep lymph nodes includes both Gram-positive bacteria such as *Staphylococcus* spp., *Streptococcus* spp., *Corynebacterium* spp., *Trueperella pyogenes* and *Rhodococcus equi*, and Gram-negative bacteria, such as *Pseudomonas aeruginosa* and *Moraxella* spp. [8–16]. Sheep infected with *Mycobacterium caprae* and *Mycobacterium bovis* can develop caseous lymphadenitis [17]. Although *M. bovis* or *M. caprae* rarely cause human tuberculosis, the importance of these pathogens should not be underestimated, especially in developing countries [18, 19] but also in other regions, cases may occur; e.g. a human case of *M. caprae* infection was recently reported in Poland [20].

Meat inspection data from 2017 to 2018 from southern Poland, especially from the Nowy Targ and Tatra counties in the Małopolska region, indicate that purulent or caseous lymphadenitis involving the mediastinal and tracheobronchial lymph nodes (MTLNs) is a common finding (slaughterhouse workers observations, unpublished data). The observed gross pathology suggests infection with mycobacteria from the *Mycobacterium tuberculosis* complex (MTBC) or *C. pseudotuberculosis*, but microbiological examination of observed lesions has not been performed. Therefore, the primary aim of the current study was to determine the aetiology of these lesions. Furthermore, it was investigated how presence of lesions was correlated with age and grazing strategy of affected sheep.

## Methods

### Animals

The animals were classified by the breeders as dual-purpose breeds i.e. used for both milk and meat production. The mean age was 78 months (6.5 years with a range from 7 to 188 months). The sheep were grouped into two based on the herd’s grazing strategy: sheep grazing locally or sheep on summer pasture in the mountains. Animals from the first group (n = 135; 4 herds) grazed on permanent pastures around the farm, where the average stocking density was nine sheep per hectare. The latter group (n = 149; 6 herds) spent the period between 1 May and 31 September grazing in the mountains. In this group, the stocking density was ten sheep per hectare.

According to data from the Agency for Restructuring and Modernization of Agriculture and the Central Statistical Office in Poland, the total sheep population of Poland in 2017 was 265,000 sheep. In the period 2016–2017, the mean number of slaughtered sheep was approximately 34,000 per year. As recommended by Thrusfield [21], the minimal sample size needed to estimate prevalence with an accepted error of 10% (level of confidence 95%, expected prevalence 50%) was 97 animals. The confidence interval (95% CI) for proportions was assessed using the Wilson score method. The calculations were performed in Win Episcope 2.0 [21].

Prior to slaughter, the sheep were identified and the documentation accompanying their consignment was checked based on printout from the herd book and food chain documentation. *Ante mortem* inspection confirmed that the animals met the requirements for slaughter. The age was determined by comparing ear tag containing an identification number with printout from herd book in which the date of birth was included. *Post*-*mortem* examination was carried out in accordance with Regulation (EC) No 854/2004 of the European Parliament and of the Council dated 29 April 2004 stating specific rules for the organisation of official controls on products of animal origin intended for human consumption (obligatory during study) [22]. This included visual inspection and palpation of the MTLNs. In case of suspected pathology, incision was made and in case of gross lesions consistent with purulent or caseous inflammation, the MTLNs were collected and frozen at − 20 °C for subsequent microbiological examination.

### Microbiological examination

After thawing, the lymphatic tissue was isolated by removing surrounding tissues and the lymph node capsule by using sterile scissors. The lymphatic tissue from each individual was then divided into two parts, from which one was homogenized with physiological solution (for conventional bacteriological study) and second with 5% oxalic acid (Sigma-Aldrich, St. Luis, MO, USA) (for mycobacterial culture) in a stomacher apparatus (MiniMix, Interscience, France) for 3 min at a frequency of 12 beats/sec in bags with side filtering membrane. The resulting suspension was poured into 50 mL tubes and subjected to accordingly conventional bacteriological examination or mycobacterial culture.

#### Conventional bacteriological examination

After homogenization in physiological solution samples were centrifuged at 3000*g* for 10 min and sediment was used for further examination. The homogenized tissue was transferred to Columbia agar supplemented with 5% (v/v) defibrinated sheep blood (Graso Biotech, Starogard Gdański, Poland) by sterile cotton swabs, streaked and cultured for 48 h at 37 °C under microaerophilic conditions. Additionally, for *R. equi* isolation, the homogenized tissues were inoculated onto a selective CAZ-NB medium (Mueller–Hinton agar base supplemented with 20 µg/mL ceftazidime and 25 µg/mL novobiocin) modified by the addition of 26 µg/mL cycloheximide and 0.005% potassium tellurite [23]. The plates were incubated for 72 h at 37 °C under aerobic conditions. After incubation, mono-cultures of isolates from each sample were prepared by streaking single colonies onto Columbia blood agar, and the bacteria were then subjected to further characterization. The isolates were identified based on their morphology, as well as their growth and biochemical characteristics. Cell morphology was visualised using Gram staining. Catalase and oxidase production were detected by conventional bacteriological methods. The API Coryne, API STAPH, API 20 STREP and API 20 E tests (bioMérieux, Craponne, France) were used to identify coryneform bacteria, staphylococci, streptococci and enterobacteria, respectively. All tests were performed according to the manufacturer’s instructions.

The CAMP test, with *S. aureus* ATCC 25923 and *R. equi* ATCC 33701 as reference strains, was performed on Columbia blood agar to determine the haemolytic activity of the coryneform isolates. The result was evaluated after 48 h incubation at 37 °C in microaerophilic conditions.

Identification of streptococci was further confirmed by 16S rRNA gene sequencing. Briefly, several colonies were suspended in 500 µL of sterile water, and boiled for 10 min at 99 °C, cooled on ice, and then centrifuged (1500*g*, 5 min). The supernatant was used as a template for polymerase chain reaction (PCR) with universal primers; UNF-5′-GAGTTTGATCCTGGCTCAG-3′ and UNR-5′-GGACTACCAGGGTATCTAAT-3′ (Genomed, Warsaw, Poland) to amplify the 16S rRNA gene fragment [14]. The reaction was carried out as follows: initial denaturation at 95 °C for 4 min, then 35 cycles at 94 °C for 30 s, 56 °C for 30 s, 72 °C for 30 s, and the final extension at 72 °C for 7 min. The PCR products were sequenced (Genomed Biotech), and BLASTn analysis of the obtained sequences was carried out at the National Centre for Biotechnology Information’s (NCBI) website (http://blast.ncbi.nlm.nih.gov).

#### Mycobacterial culture

The homogenates for mycobacterial culture were incubated for 15 min at 37 °C. The samples were then centrifuged at 3000*g* for 10 min, the supernatant was discarded and a sterile 0.9% NaCl solution (Polfa-Lublin S.A., Lublin, Poland) was added to the sediment to fill the tube volume. The tubes were shaken and centrifuged again as above. The procedure was repeated twice to effectively remove the oxalic acid, and then the supernatant was discarded and the sediment used to set up cultures on solid media: Löwenstein-Jensen and Stonebrink (Becton Dickinson, Franklin Lakes, NJ, USA). The cultures were incubated at 37 °C and checked for mycobacterial growth every 7th day for 12 weeks. If no growth was observed after 12 weeks, the sample was considered negative.

### Statistical analysis

A binary logistic regression model was used to determine whether any of the recorded factors (age, grazing type) influenced the presence of purulent or caseous lymphadenitis in the MTLNs. The presence of bacteria, regardless of the species, was used as a response variable. Age and grazing type were covariates. Age was a linear variable expressed as months of life. Grazing type was a grouping factor. The herds were not compared as separate groups in the model because some were underrepresented, i.e. with a small sample size. For the same reason, i.e. rare occurrence of individual bacterial species, separated models were not constructed for the occurrence of particular bacterial species. The statistical analysis was performed using SPSS software (version 24.0, IBM Corporation, Armonk, NY, USA).

## Results

### *Post*-*mortem* examination

**Fig. 1:**
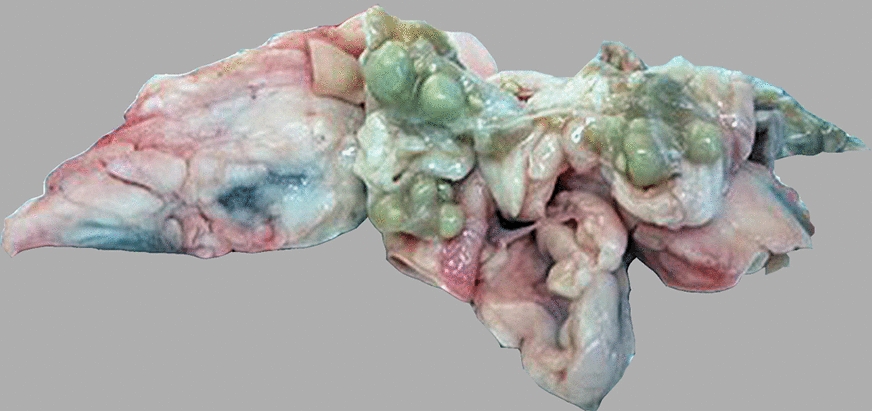
Enlarged and firm thoracic lymph nodes with purulent lymphadenitis from a slaughtered sheep

Purulent or caseous lymphadenitis was detected in MTLNs in 49 out of 284 carcasses (17.3%; CI 95%: 13.3–22.1%). Most of these affected lymph nodes were enlarged (Fig. 1) and contained firm, compact, dark yellow to green, usually mineralised pus.

### Microbiological examination

Bacteria were cultured from all lymph nodes with lymphadenitis but pathogenic bacteria were isolated only from 39 lymph node samples, mainly as mono-cultures (Table 1). In two samples, two both *C. pseudotuberculosis* and *Streptococcus dysgalactiae* subsp. *equisimilis* were isolated indicating co-infections. Presence of bacteria in the other lymph node samples (n = 10) were considered as due to contamination.

**Table 1:** Prevalence of pathogenic bacteria in ovine mediastinal and tracheobronchial lymph nodes (n = 49) with purulent or caseous lymphadenitis

Bacteria	Number of isolates (%)
*Corynebacterium pseudotuberculosis*	17* (34.7%)
*Streptococcus dysgalactiae* subsp*. equisimilis*	17** (34.7%)
*Staphylococcus aureus*	4 (8.2%)
*Trueperella pyogenes*	1 (2.0%)
*Escherichia coli*	1 (2.0%)
*Enterococcus* spp.	1 (2.0%)

* Co-infection with *S. dysgalactiae* subsp. *equisimilis* in two cases

**Co-infection with *C. pseudotuberculosis* in two cases

Seventeen isolates of bacteria considered as the cause of lymphadenitis were Gram-positive irregular rods having small, waxy, cream-white colonies with a narrow zone of ß-haemolysis which is typical for *Corynebacterium* spp. For all these isolates, the CAMP test revealed enhanced haemolysis in the presence of *R. equi* ATCC 33701 but inhibited haemolysis for the *S. aureus* ATCC 25923 culture. This result being typical for *C. pseudotuberculosis*, was confirmed by the API Coryne test (92.8–99.6% of identification). The nitrate reduction tests were negative, thus classifying all isolates as *C. pseudotuberculosis* biovar *ovis* [24].

Other 17 samples contained ß-haemolytic streptococci, identified on the basis of cell and colony morphology. The isolates demonstrated variable biochemical profiles and therefore underwent 16S rRNA gene sequencing: subsequent BLASTn analysis indicated 99% shared identity with *S. dysgalactiae* subsp. *equisimilis*.

*Staphylococcus aureus* was the suspected cause of inflammation in four lymph node samples, while *T. pyogenes*, ß-haemolytic *Escherichia* coli and an *Enterococcus* spp. were the cause in one case each. *R. equi* or mycobacteria were not isolated.

### Effects of age and grazing type on the occurrence of purulent or caseous lymphadenitis

The regression model correctly classified over 82% of all cases. The model indicated that both age and grazing type affected the probability of lesions being present in the MTLNs (Table 2). In older sheep, the risk of developing lymphadenitis in the MTLNs was higher than in younger, the risk increased by 1.5% with each month of life. Sheep grazing locally had 4.5-times greater risk of having lymphadenitis than individuals grazing in the mountains during summer.

**Table 2:** Effects of age and grazing type on the occurrence of purulent or caseous lymphadenitis in the mediastinal and tracheobronchial lymph nodes of sheep

Source	*B*	*Waldχ*^2^	*Df*	*P*	*ODDS*
Intercept	− 3.621	43.46	1	< 0.001	0.027
Age	0.015	11.80	1	0.001	1.015
Grazing (local)	1.504	15.74	1	< 0.001	4.500

Binary logistic regression model (χ^2^ = 22.14; df = 2; P < 0.001), all predictors statistically significant. *B* regression coefficient, *Wald χ*^*2*^ Wald Chi Squared Test of significance of each predictor, *Df* degrees of freedom, *P* calculated probability, *ODDS* odds ratio-the exponentiation of the B coefficient (exp(B))

## Discussion

The bacteria isolated from purulent or caseous lesions in the MTLNs may be zoonotic. These include *C. pseudotuberculosis* [25, 26], *S. aureus* [27, 28], *S. dysgalactiae* subsp. *equisimilis* [29, 30], or *T. pyogenes* [31]. Slaughterhouse workers, veterinarians or other industry employees who have direct contact with infected tissues are particularly exposed to infections with these microorganisms.

The frequent isolation of *C. pseudotuberculosis* from the MTLNs indicates that this pathogen can have influence on small ruminant health, which had been confirmed in previous reports [32, 33]. In the last few decades there has been an increasing number of caseous lymphadenitis (CLA) cases, caused by *C. pseudotuberculosis*, in sheep and goat flocks in many regions of the world [9, 34, 35]. CLA is manifested as an abscesses containing white-yellow dense pus often with mineralization. In the progressive stages the lesion appears lamellated resembling an onion after cutting (pathognomonic signs of CLA) [36]. In sheep, the lesions are mainly located in the lungs and MTLNs [36]. CLA transmission is being facilitated by movement of infected animals between herds [32] and transmission can be either direct (physical contact) or indirect (surfaces, feed, water, soil contaminated with fomites). Due to difficulties in detecting asymptomatic shedders, and the ability of bacteria to survive in the environment, the disease can be endemic in large herds of small ruminants [32, 37]. Although CLA-related mortality is relatively low among sheep and goats, the disease nevertheless entails significant economic losses associated with poor health, lowered wool quality, decreased meat and milk production and prolongation of *post*-*mortem* inspection [8, 38–41]. Further research in on CLA in Polish small ruminants seems justified, as CLA-associated lymphadenitis was a common finding.

Our findings confirm that *S. dysgalactiae* subsp. *equisimilis*, which also causes purulent lymphadenitis [42], was abundantly present in the examined sheep. Only a limited amount of literature currently exists on such infections of sheep, though Rutherford et al. [43] demonstrated the presence of *S. dysgalactiae* in arthritis of lambs. Also, only single studies describe *S. aureus* in small ruminants [28]. *T. pyogenes*, a bacterium responsible for purulent infections and organ abscesses, including purulent pneumonia in sheep [44] was also isolated in this study, but only in one case.

*Rhodococcus equi* was not isolated from the examined sheep, confirming previous studies reporting low prevalence in ruminants and hence *R. equi* seems to be of minor importance [45]. In Poland, to date, this species has been detected in the lymph nodes of clinically healthy cattle, red deer (*Cervus elaphus*) and roe deer (*Capreolus capreolus*) [16, 46]. However, *R. equi* should still be considered a potential pathogen of sheep, because it has been isolated from domestic goats (*Capra hircus*), camels (*Camelus dromedarius*), and llamas (*Lama glama*) in other countries [47]. Moreover, *R. equi* has been detected in the lymph nodes of cattle and American bison (*Bison bison*) in co-infection with *Mycobacterium* spp. [48].

Mycobacteria were not isolated from any of the examined cases. Sheep are generally considered less susceptible to tuberculosis than other ruminants [49]. If infected, sheep may develop lesions in the lungs and MTLNs [17]. Poland has officially been declared a free from bovine tuberculosis due to *M. bovis* in accordance with EU Commission Decision 2009/342/EC of 23 April 2009 [50]. However recently, cases of bovine tuberculosis have been diagnosed in the studied area [51]. Veterinary abattoir inspections were governed by Regulation (EC) No 854/2004 of the European Parliament and Council [22] during the study period. This regulation specifies that when tuberculosis-like lesions, i.e. purulent to caseous lymphadenitis occur, a detailed examination of the carcass should be performed to check for gross lesions in other lymph nodes and organs. If no additional lesions are detected, only the organ system with the affected lymph nodes should be condemned, and the rest of the carcass is declared fit for human consumption. Small ruminants are not included in the bovine tuberculosis *ante*-*mortem* control programme in Poland. The procedure of *post*-*mortem* examination of the thoracic region of sheep and goats includes a visual inspection of the trachea, oesophagus and lungs, as well as palpation of the lungs and the MTLNs. The organs and lymph nodes are only to be incised and examined if visual inspection and palpation reveal abnormalities. However, it is doubtful if all lesions, which potentially pose a risk to consumers, will be detected during visual examination or palpation. Therefore, the existing *post*-*mortem* inspection procedure may be insufficient, and its ability to ensure protection of public health may require reassessment and the presence of pulmonary lymphadenitis is sheep should raise differential diagnosis concerns despite sheep’s low susceptibility to *M. caprae* and *M. bovis*.

The epidemiological analyses indicate a much higher risk of having lymphadenitis in the MTLNs of older sheep; a finding probably associated with a greater likelihood of contact with infectious agents in longer-lived sheep. A significant difference in the incidence of lymphadenitis was also observed with regard to grazing strategy, where lesions were found 4.5 times more frequently in sheep grazing on permanent pastures compared to mountain pastures. This may be due to exposure to a larger number of pathogens (higher infection pressure) on permanent pastures as bacteria may survive in the environment from one grazing season to the next. Differences in hygiene conditions, such as indoor temperature and humidity or stocking density, between the two populations may also affect the frequency of aerogenic infections, which are of particular importance for the development of pulmonary CLA [52].

## Conclusions

A high prevalence of purulent or caseous lymphadenitis in the MTLNs due to potentially zoonotic bacteria was found in sheep in Małopolska region of Poland. The most prevalent bacteria were *C. pseudotuberculosis* and *S. dysgalactiae* subsp. *equisimilis*. Particular attention during *post*-*mortem* examination should be paid to the carcasses of older sheep and sheep grazing on permanent pastures, as they seem more prone to develop pulmonary lymphadenitis.

## Data Availability

All data and materials are available at the Department of Food Hygiene and Public Health Protection, Institute of Veterinary Medicine, Warsaw University of Life Sciences–SGGW, ul. Nowoursynowska 159, 02-776 Warsaw, Poland

## Abbreviations

*B*: Regression coefficient
CLA: Caseous lymphadenitis
*Df*: Degrees of freedom
MTBC: *Mycobacterium tuberculosis* complex
MTLNs: Mediastinal and tracheobronchial lymph nodes
*ODDS*: Odds ratio (Exp(B))
*P*: Calculated probability
PCR: Polymerase chain reaction
*Wald χ*^*2*^: Wald Chi-Squared Test of significance of each predictor
